# Designing Novel Multi-Epitope Vaccine Construct against *Prevotella intermedia*-Interpain A: An Immunoinformatics Approach

**DOI:** 10.3390/medicina59020302

**Published:** 2023-02-06

**Authors:** Pradeep Kumar Yadalam, Raghavendra Vamsi Anegundi, Safa Munawar, Ramya Ramadoss, Santhiya Rengaraj, Sindhu Ramesh, Mohammed Aljeldah, Basim R. Al Shammari, Ahmad A. Alshehri, Ameen S. S. Alwashmi, Safaa A. Turkistani, Abdulsalam Alawfi, Amer Alshengeti, Mohammed Garout, Amal A. Sabour, Maha A. Alshiekheid, Fatimah S. Aljebaly, Ali A. Rabaan

**Affiliations:** 1 Department of Periodontics, Saveetha Institute of Medical and Technical Sciences, Saveetha Dental College, Saveetha University, Chennai 600077, India; 2 Department of Medical Education, Nawaz Sharif Medical College, Gujrat 50700, Pakistan; 3 Department of Oral Pathology & Oral Biology, Saveetha Institute of Medical and Technical Sciences, Saveetha Dental College and Hospitals, Saveetha University, Chennai 600077, India; 4 Adhiparasakthi Dental College and Hospital, Melmaruvathur, Chennai 600077, India; 5 Department of Conservative Dentistry and Endodontics, Saveetha Institute of Medical and Technical Sciences, Saveetha Dental College, Saveetha University, Chennai 600077, India; 6 Department of Clinical Laboratory Sciences, College of Applied Medical Sciences, University of Hafr Al Batin, Hafr Al Batin 39831, Saudi Arabia; 7 Department of Clinical Laboratory Sciences, College of Applied Medical Sciences, Najran University, Najran 61441, Saudi Arabia; 8 Department of Medical Laboratories, College of Applied Medical Sciences, Qassim University, Buraydah 51452, Saudi Arabia; 9 Fakeeh College for Medical Science, Jeddah 21134, Saudi Arabia; 10 Department of Pediatrics, College of Medicine, Taibah University, Al-Madinah 41491, Saudi Arabia; 11 Department of Infection Prevention and Control, Prince Mohammad Bin Abdulaziz Hospital, National Guard Health Affairs, Al-Madinah 41491, Saudi Arabia; 12 Department of Community Medicine and Health Care for Pilgrims, Faculty of Medicine, Umm Al-Qura University, Makkah 21955, Saudi Arabia; 13 Department of Botany and Microbiology, College of Science, King Saud University, Riyadh 11451, Saudi Arabia; 14 Department of Basic Medical Sciences, Unaizah College of Medicine and Medical Sciences, Qassim University, Buraydah 51452, Saudi Arabia; 15 Molecular Diagnostic Laboratory, Johns Hopkins Aramco Healthcare, Dhahran 31311, Saudi Arabia; 16 College of Medicine, Alfaisal University, Riyadh 11533, Saudi Arabia; 17 Department of Public Health and Nutrition, The University of Haripur, Haripur 22610, Pakistan

**Keywords:** periodontitis, biofilm, red complex, bone loss, public health

## Abstract

*Background and Objectives*: Periodontitis is a chronic multifactorial inflammatory infectious disease marked by continuous degradation of teeth and surrounding parts. One of the most important periodontal pathogens is *P. intermedia*, and with its interpain A proteinase, it leads to an increase in lethal infection. *Materials and Methods*: The current study was designed to create a multi-epitope vaccine using an immunoinformatics method that targets the interpain A of *P. intermedia*. For the development of vaccines, *P. intermedia* peptides InpA were found appropriate. To create a multi-epitope vaccination design, interpain A, B, and T-cell epitopes were found and assessed depending on the essential variables. The vaccine construct was evaluated based on its stability, antigenicity, and allergenicity. *Results:* The vaccine construct reached a more significant population and was able to bind to both the binding epitopes of major histocompatibility complex (MHC)-I and MHC-II. Through the C3 receptor complex route, *P. intermedia* InpA promotes an immunological subunit. Utilizing InpA-C3 and vaccination epitopes as the receptor and ligand, the molecular docking and dynamics were performed using the ClusPro 2.0 server. *Conclusion*: The developed vaccine had shown good antigenicity, solubility, and stability. Molecular docking indicated the vaccine’s 3D structure interacts strongly with the complement C3. The current study describes the design for vaccine, and steady interaction with the C3 immunological receptor to induce a good memory and an adaptive immune response against Interpain A of *P. intermedia*.

## 1. Introduction

With the advances in technology and science, significant progress has been made in understanding microorganisms and disease processes. The knowledge of the disease process has undergone a paradigm shift from early models that assumed the quantity of plaque to current concepts of host-microbial interactions [1]. Periodontitis is a microbial-induced, host-mediated, chronic inflammatory disease characterized by dysbiotic plaque biofilms that cause progressive attachment loss [2,3]. Some bacteria, even in small quantities, can interact with the host’s immune system and other bacteria, increasing the pathogenicity of the microbiome. The dysbiotic microbiota includes a variety of microorganisms, of which *Prevotella intermedia*, *Porphyromonas gingivalis*, and *Aggregatibacter actinomycetemcomitans* have significant effects on the microbiota, disrupting tissue homeostasis [4,5]. 

A localized chronic inflammation and subsequent loss of the tooth’s supporting structures result from the intricate interplay of the bacterial virulence factors and defense mechanisms of the host [6,7]. Many periodontal pathogens create proteinases, which are key virulence factors that can lead to the host’s proteins being broken down for vital nutrients. Many strains of *P. intermedia* have evolved defenses against complement killing to become successful pathogens [8]. The function of the complement system is to promote the uptake and destruction of pathogens by phagocytic cells. Complement receptors (CRs) on phagocytes detect bound complement components. These complement receptors bind pathogens that have been opsonized by complement proteins, which is one of the main roles of C3b and its proteolytic derivatives. Since C3b is produced in greater quantities than C4b, C4b also functions as an opsonin but plays a considerably smaller role. Hence, microbes have found ways to evade this system for better survival. Similar to how gingipains have favoured the chain of C3 and C4, Interpain A (InpA) also demonstrated this preference. Gingipains primarily bind the chains of these proteins, allowing them to release the complements C3a and C5a, which serve as anaphylatoxins, and their induced versions C3b, C4b, and C5b at low doses [9].

*Prevotella intermedia*, an oral Gram-negative anaerobe, helps in converting hemoglobin to an iron (III) protoporphyrin IX pigment. The bacterium produces InpA (interpain A), a 90-kDa cysteine protease, a homolog of streptopain from *Streptococcus pyogenes* (SpeB). Under situations of low redox potential and higher pH in the infected gingival crevice and diseased periodontal pocket, where the host closely regulates the availability of haeme, InpA greatly contributes to the acquisition of haeme [10]. Haem albumin is more sensitive to InpA than apo albumin. In order to extract haemoglobin’s hemosiderin from the cell, *Prevotella intermedia*’s cysteine protease Interpain A (InpA) collaborates with *P. gingivalis*’ HmuY hameophore, demonstrating further post-translational interaction. Notably, gingipains work in concert with karilysin or Interpain A to suppress complement, indicating that these complementary proteases may still prevent complement activation after being released and diffused across the biofilm that is defending the entire microbial community [11].

Computational immunology, often known as immunoinformatics, is a subfield of bioinformatics that uses bioinformatics methods to comprehend and analyze immunological data [12]. Utilizing databases and other technologies to predict B- and T-cell epitopes is one of the most researched aspects of applied immunology. Researchers can now utilize an organism’s genome information to identify vaccine candidates computationally, moving beyond the old vaccinology method thanks to advancements in sequencing tools [13,14]. Major colonization factors, adhesion proteins, and other well-characterized virulence components essential for infection initiation and additional host damage have been the main targets of vaccine development. The pathogen genome does, however, encode several uncharacterized proteins that have yet to be investigated for their potential to encode antigenic regions. Immunoinformatics techniques can be beneficial, especially for diseases with minimal information on pathogenesis mechanisms or antigenic determinants [15,16].

Since *P. intermedia* (Interpain A) has a significant role in host immune modulation and providing the required nutrition to the microbiome, the current study aimed to design a vaccine that targets Interpain A using an immunoinformatics approach.

## 2. Materials and Methods

### 2.1. Sequence Analysis

Immune epitope database analysis was used to identify the protein structure of the epitope of *P. intermedia* with the help of positive assays for linear epitopes [17]. The network assembled was examined for hubs, shortest path, and clustering coefficient. The Protein Data Bank (PDB) database was used to retrieve the amino acid (FASTA) reference identity (ID) of 3BBA, which belongs to *P. intermedia*. The antigenic peptides prediction tool (http://imed.med.ucm.es/Tools/antigenic.pl) (accessed on 12 March 2022) and the AllerTop v2.0 servers (http://ddg-pharmfac.net/AllergenFP/) (accessed on 12 March 2022) were used to screen 3BBA for average antigenic propensity and allergenicity [12].

### 2.2. Prediction of Epitope

Using NetCTL1.2 (DTU Health Tech, Lyngby, Denmark) [18], lymphocyte (CTL-cytotoxic-T cells) epitopes for 3BBA were predicted for serotypes that had a threshold value of 0.75, 0.97 (specificity), or 0.80 (sensitivity). Default levels of C-terminal cleavage and transporter associated with the antigen were used. Both immune and antigen reactivity were ascertained using Class-I immunogenicity of the IEDB server and VaxiJen v2.0. Using a traditional method and a percentile rank of 2, the MHC-I specific gene sequence of a subset of CTL epitopes (17 epitopes/ligands) was found with MHC-I related predictions in the Immune Epitope Database (http://tools.iedb.org/mhci/), (accessed on 14 March 2022). The percentile rank and IC50 value of peptide-MHC-II interactions were determined using the NN Align technique and the IEDB MHC-II epitope prediction tool. The origin species was a person. Further analysis was conducted on the Human Leukocyte Antigen–DR isotype (HLA-DR), HLA-DP, and HLA-DQ loci. Since these results represent a greater affinity, IC50 values of 10 nM and a percentile rank of 1.5 were utilized for prediction. We assessed the antigenic properties of anticipated HTL epitopes. Finally, the allergenicity, toxicity, and antigenicity of the 3BBA epitopes from Cytotoxic T lymphocytes (CTL), Helper T cell (HTL), and B cell lymphoma (BCL) were considered. For the creation of multi-epitope vaccines, the predicted 3BBA epitopes were utilized [13]. 

### 2.3. Population Coverage Analysis

Expression and distribution of HLA alleles expressed diverse presentations between regions and ethnicity, which may impact the creation of multi-epitope-based vaccines [19]. The population coverage tool of IEDB was used to assess the CTL and HTL.

### 2.4. Construction of Multi Epitope Vaccine

Adjuvant, CTL, HTL, and BCL epitopes were combined to form appropriate links to allow the epitopes, in vivo, adequate room to function. Human-defensin-2 (PDB ID: 1FD 3) served as an adjuvant along with a B-cell epitope utilizing an EAAAK linker to boost the immunogenicity of the vaccine candidate [14,20]. The same GSGSGS, GSGSGS, and AAY linkers were used to bind BCL to HTL, HTL to CTL, and intra-CTL epitopes, respectively.

### 2.5. Structure Prediction and Validation

Through the use of the Swiss model, the iterative threading modeling method was used to predict and validate the three-dimensional structure of the vaccine construct. 

### 2.6. Molecular Docking Analysis

Utilizing 2a73 complement C3 and the vaccine receptor and ligand, molecular docking with ClusPro 2.0 program was used in order to assess the co-action of the vaccine and with the host immune receptor. So, using three sequential steps—rigid body docking, clustering of lowest energy structures, and structural refinement—complexes were created. The docked structure was examined using PyMol (http://www.pymol.org) (accessed on 15 March 2022), and the ideal complex was selected to assess which complex had a lesser energy score [12,13].

### 2.7. Molecular Dynamics Simulation

The dynamics and structural stability of protein complexes are effectively investigated by molecular dynamics (MD) simulation. Desmond software (D. E. Shaw Research, New York, NY, United States) used MD to imitate vaccine-C3. Desmond (Schrödinger LLC, New York, NY, USA) ran a 100-nanosecond simulation of molecular dynamics. The receptor-ligand complex was reduced and optimized by Maestro’s Protein Preparation Wizard. Using System Builder, all systems were created. An orthorhombic solvent model is TIP3P. 

(Points from TIIP3) The simulation made use of OPLS 2005. NaCl at 0.15 M simulated physiological conditions. The ensemble of 300 K and 1 atm NPT was used throughout the simulation. Simulation models were lax. The simulation’s stability was evaluated by comparing the root-mean-square deviation (RMSD) over time for the protein and ligand.

## 3. Results

### 3.1. Analysis of P. intermedia Peptide Sequences

**Figure 1:**
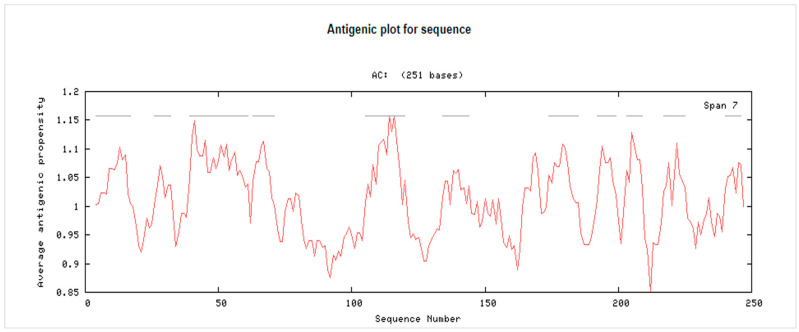
The antigenic propensity of reference ID 3BBA. AC: Amino acids.

The molecular weight of the peptide 3BBA was approximately 251 amino acids bases. In vaccine development, epitope identification assesses proteins whose antigen prediction was higher than 0.8 (Supplementary Figure S1). The antigenic susceptibility of 3BBA was found to be 1.0122 on average, and they were not allergic (Supplementary Figure S2). For the creation of a multi-epitope vaccine against *P. intermedia*, 3BBA was chosen based on its antigenic propensity (Figure 1 and Supplementary Figure S3).

### 3.2. Prediction and Assessment of T-Lymphocyte Epitope 

Cytotoxic T-lymphocyte epitopes are essential for eliciting robust immune reactions involving the histocompatibility complex. The NetCTL1.2 service was used to find the epitopes of 3BBA. Fifteen epitopes from 3BBA with cumulative scores greater than 0.75 were discovered from all MHC-I serotypes (Table 1 and Supplementary Table S1). Helper T-lymphocytes activate cytotoxic T-cells to produce antibodies and kill infected target cells. For the HLA-DR, HLA-DQ, and HLA-DP loci, HTL epitopes for 3BBA were predicted using IC50 values (10 nM) and percentile rank (1.5). The HTL epitopes at the HLA-DR gene met several requirements. The HTL epitope (obtained from MHC-II) was discovered to be similar. The C-terminal dimerization domain employed in the development of vaccines was chosen to contain the HTL epitope (LAEVKALTTELTAEN) (Table 2).

### 3.3. Prediction and Assessment of B-Lymphocytes

**Figure 2:**
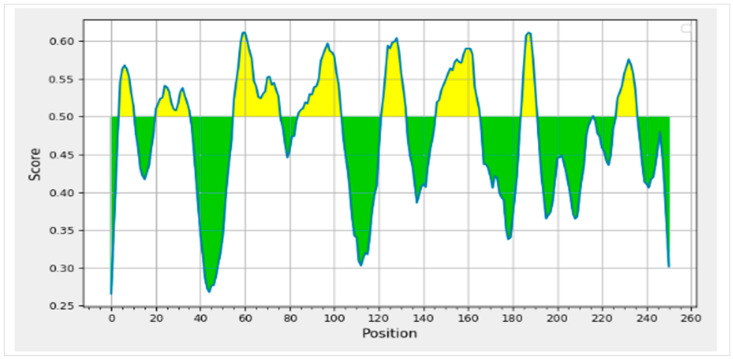
Graphical representation of the BCL epitopes’ confirmation with the BepiPred server identified by ABCPred with a 16-mer length score of 0.5 or above.

B-cell epitopes are crucial in the production of antibodies. The BepiPred server confirmed the B-cell epitopes identified by ABCPred (Table 3) with a 16-mer length score of 0.5 or above (Figure 2). The BCL epitopes for 3BBA were discovered to satisfy server requirements by demonstrating antigenic, non-allergic, and non-toxic characteristics (Table 4 and Table 5). Ultimately, 3BBA was chosen for vaccine production based on the study and prediction of CTL, HTL, and BCL.

### 3.4. Analysis of Population Coverage

**Figure 3:**
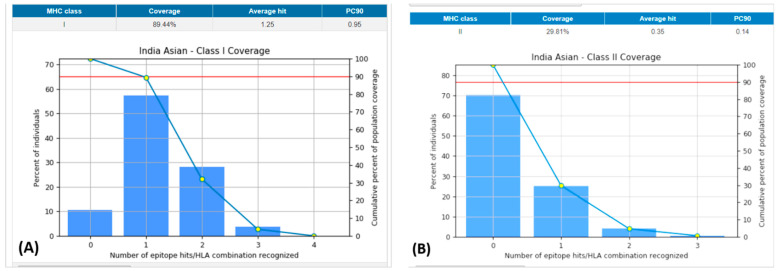
Population coverage of all the selected epitopes for both MHC-I and II classes. (**A**): Indian Asian class-I coverage. (**B**): Indian Asian class-II coverage. MHC: major histocompatibility complex.

The population coverage of the peptide epitope 3BBA from *P. intermedia* was examined (Table 6). The combined MHC-I and MHC-II epitopes revealed 89.44% and 29.81% of population coverage worldwide, respectively, using the chosen T-cell epitopes with cognate HLA alleles (Figure 3). Notably, our vaccine candidate has demonstrated wider population coverage to combat *P. intermedia* globally.

### 3.5. Construction of Multi-Epitope Vaccine

The epitopes chosen from the top-scoring MHC classes 1 and 2 were stitched together by appropriate linkers to create a multi-epitope vaccine. An EAAAK linker was used to bind the adjuvant to the BCL epitope to prevent adjuvant interaction with the vaccine design. An AAY linker was used to unite CTL epitopes, maintaining the structural configuration of the epitopes while increasing the likelihood of antigenic reactivity. Additionally, BCL to HTL and HTL to CTL epitopes were linked together using the linker GSGSGS, which gives proteins structural flexibility without affecting the function of vaccine candidates.

### 3.6. Molecular Docking

**Figure 4:**
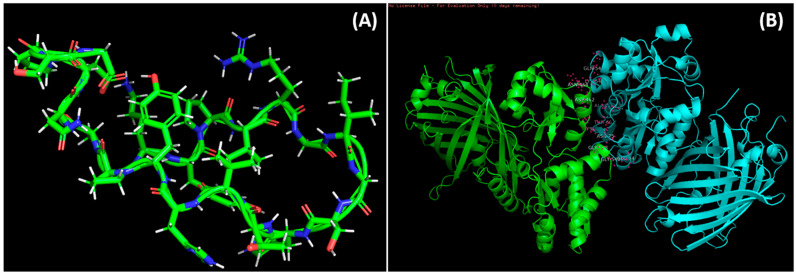
Docked complexes of the vaccine and the epitopes. (**A**): Predicted structure of modeled vaccine construct using a Swiss model. (**B**): Molecular docking of epitopes with 2a37 complement c3 receptor.

ClusPro 2.0-based molecular docking was conducted to evaluate the vaccine components’ interactions with Human Complement Component C3 PDB id-2a73. The best complex of vaccine-C3 was preferred as it presented the lowest energy score (596.9 kJ mol^−1^) and center energy (the energy between receptor and ligand) of −661.2 kJ mol^−1^ (Supplementary Figure S4). The vaccine candidate’s residues displayed polar contact with the C3 receptor residues (Figure 4B). The Molecular Dynamic Simulation Analysis has been shown in Supplementary Figure S5.

The stability and compactness of the docked vaccine-C3 complex were investigated using the Desmond tool and a 100 ns molecular dynamics simulation. The complicated RMSD (1) plot demonstrates that stability was obtained at 40 ns. Following that, fluctuations in protein RMSD values remained within 1.5 throughout the simulation time. The RMSD values of ligands range from 2.0 angstrom to 100 ns.

## 4. Discussion

The primary goal of the periodontal vaccine is to depreciate disease progression and eventually eradicate periodontal disease [21]. *P. intermedia* is a bacterium that is closely associated with various periodontal diseases and other infections [22,23]. *P. intermedia* has frequently been found in subgingival plaque in human patients with necrotizing gingivitis, pregnancy gingivitis, and adult periodontitis [24]. Additionally, *P. intermedia* is challenging to eliminate because it quickly develops antibiotic resistance [25]. An in-depth molecular understanding of infection and resistance is essential for developing alternative treatments [26]. Numerous proteases, including trypsin-like serine proteases, dipeptidyl peptidase IV, and cysteine proteases, have been identified in *P. intermedia* [27]. However, the development of structural studies enabled us to comprehend their unique mode of action and aid in designing vaccines. Epitope-based vaccines have frequently been created using immunoinformatics, an innovative and practical approach [28].

Interpain A, a cysteine protease from the cysteine-histidine-dyad class, was investigated in its zymogenic and mature self-processed forms. The latter is made up of a bivalved portion with two subdomains. Complement is an important component of the innate immune defense system, with the primary purpose of recognizing and killing bacteria [28,29].

Heat inactivation of the complement system greatly reduces the opsonic activity in vitro, indicating that complement is required for host defense against *P. intermedia* [30,31]. Moreover, in the absence of the classical system, the alternative pathway opsonized *P. intermedia*, most likely due to a reaction to endotoxin. Yet, kinetic tests demonstrated that opsonization occurred substantially faster when the classical pathway was intact [32]. Surprisingly, the alternative pathway contributed to the death of serum-sensitive strains, whereas the traditional pathway was predominantly responsible for the death of intermediate-sensitivity strains [33]. As a result, the complement appears to recognize *P. intermedia* through many sensory chemicals. However, it appears that *P. intermedia* can bind to C3 and, to some extent, overwhelm complement defenses, allowing chronic infections to develop in the oral cavity [34]. Furthermore, Interpain A acts synergistically with *P. gingivalis* gingipains [35]. When applied in equal quantities, C3b deposition was reduced by 85%, compared to 55% when added individually. Moreover, combining three gingipains with InpA reduced the C3b deposition by 93%. Hence, the C3 target used in the current study is relevant and practical since the multiple vaccine epitope inhibits the binding of Interpain A to C3, besides eliminating the interaction or association with other periodontopathogens [27].

Epitopes induce cytotoxic T- and B-cell lymphocytes to destroy pathogenic microbes through cytokine action [36]. Cytokines utilize helper T-lymphocytes to trigger the immune system. The *P. intermedia* 3BBA epitope peptide depicted higher antigenicity, immunogenicity, non-allergenic and non-toxic nature, and increased MHC-I and II binding than other epitopes. The present study results show that the current vaccine design is non-allergic, non-antigenic, non-toxic, and does exhibit good immunogenicity. Moreover, molecular docking and dynamics results exhibit excellent and stable binding throughout the stimulation period. This is due to the increased affinity of the vaccine for the C3 receptors. Further studies with DNA cloning are required to make this vaccine a reality. 

## 5. Conclusions

The current study provides the first vaccine design for Interpain A using an Immunoinformatic approach. *P. intermedia* is a common bacterium that is associated with periodontal infections. It also contributes to the sustainability of the microbiome by providing essential substrates such as albumin and haem. Future well-designed studies are required to evaluate the efficacy of this vaccine design. Furthermore, it will be interesting to observe the overall effect of the elimination of *P. intermedia* on the microbiome.

## Figures and Tables

**Table 1:** MHC-I binding predictions of IEDB server.

Allele	#	Peptide *	Core	Score	Score	Percentile Rank
HLA-A * 01:01	6	SADFGNTTY	SADFGNTTY	SADFGNTTY	0.976649	0.01
	2	TTWGQQMPY	TTWGQQMPY	TTWGQQMPY	0.634927	0.12
	13	LTKGHPLIY	LTKGHPLIY	LTKGHPLIY	0.631891	0.12
	3	TATAQVLNY	TATAQVLNY	TATAQVLNY	0.466786	0.21
	15	EQDMVRGVY	EQDMVRGVY	EQDMVRGVY	0.46399	0.21

* Starts at 1, ends at 9, and the total length was 9. #: Number.

**Table 2:** MHC-II binding peptides (Consensus (comb.lib./smm/nn)) accessed on 17 March 2022.

Allele	#	Start	End	Peptide *	Percentile Rank	Adjusted Rank
HLA-DRB1 * 01:01	1	55	69	FKYPVRGIGSHTVHY	8.40	8.40
	1	98	112	SGNYTEAEANAVATL	8.70	8.70
	1	99	113	GNYTEAEANAVATLM	8.90	8.90
	1	6	20	PSKYAAEVSTLLTTT	8.90	8.90
	1	97	111	YSGNYTEAEANAVAT	9.70	9.70

* Total length was 15. #: Number.

**Table 3:** Identification of B-cell epitopes (ABCPred).

Rank	Sequence	Start Position	Score *
1	VRGIGSHTVHYPANDP	59	0.93
2	DFGNTTYDWANMKDNY	82	0.90
3	HPLIYGGVSPGSMGQD	176	0.87
3	SGAYMTDCAAGLRTYF	131	0.87
4	SGTAISADFGNTTYDW	75	0.86
5	GGPNEGSGAYMTDCAA	125	0.84

* A higher peptide score depicts a probable epitope.

**Table 4:** Predicted peptides.

No.	Start	End	Peptide	Length
1	5	11	DPSKYAA	7
2	21	36	WGQQMPYNKLLPKTKK	16
3	56	76	KYPVRGIGSHTVHYPANDPSG	21
4	85	104	NTTYDWANMKDNYSGNYTEA	20
5	122	133	MQYGGPNEGSGA	12
6	147	166	GFTDAEYITRANYTDEQWMD	20
7	185	192	PGSMGQDA	8
8	217	217	V	1
9	228	236	PGNMYSFTA	9

**Table 5:** Toxicity prediction of epitopes.

Peptide Sequence *	SVM Score	Hydrophobicity	Hydropathicity	Hydrophilicity	Charge
YAAEVSTLL	−1.36	0.12	1.01	−0.61	−1.00
TTWGQQMPY	−1.25	−0.11	−1.19	−0.82	0.00
TATAQVLNY	−0.91	−0.01	0.21	−0.78	0.00
ATAQVLNYF	−0.90	0.08	0.60	−1.01	0.00
GIGSHTVHY	−0.84	0.04	−0.14	−0.74	1.00
SADFGNTTY	−0.93	−0.10	−0.70	−0.29	−1.00
MKDNYSGNY	−0.90	−0.32	−1.81	0.09	0.00
YTEAEANAV	−0.99	−0.08	−0.32	0.06	−2.00
MTDCAAGLR	−0.78	−0.13	0.30	0.06	0.00
FTDAEYITR	−0.48	−0.21	−0.57	0.12	−1.00
YTDEQWMDI	−1.28	−0.17	−1.17	0.00	−3.00
WMDIVFSEL	−1.43	0.16	0.94	−0.67	−2.00
LTKGHPLIY	−1.28	0.02	0.11	−0.62	1.50
WNGDVDGYY	−0.50	−0.09	−1.18	−0.37	−2.00
EQDMVRGVY	−1.42	−0.25	−0.71	0.29	−1.00

* non-toxin.

**Table 6:** India Asian-class II coverage.

Number of HLA Combinations Identified	Percent of Individuals	Cumulative Percent of Population Coverage (in %)
0	70.19	100
1	25.08	29.81
2	4.06	4.72
3	0.66	0.66

HLA: human leukocyte antigen.

## Data Availability

Not applicable.

## Supplementary Materials

The following supporting information can be downloaded at: https://www.mdpi.com/article/10.3390/medicina59020302/s1, Figure S1: Antigenic Peptides determinants; Figure S2: Prediction of Allergenicity; Figure S3: Antigenicity Prediction; Figure S4: Binding energy of the Predicted vaccine structure with C3; Figure S5: Molecular dynamic simulation analysis; Table S1: CTL epitopes for N protein (NetCTL1.2).
