# Supplementary material for: Designing Novel Multi-Epitope Vaccine Construct against Prevotella intermedia-Interpain A: An Immunoinformatics Approach
Source: Medicina (Kaunas). 2023 Feb 6;59(2):302. doi: 10.3390/medicina59020302 (PMC9966096; doi:10.3390/medicina59020302)
Supplement: Supplementary file 1 [file medicina-59-00302-s001.zip › medicina-2061227-supplementary.pdf]

n	Start Position	Sequence	End Position
1	4	PDPSKYAAEVSTLL	17
2	26	PYNKLLP	32
3	39	LITGCVATATAQVLNYFKYPVRG	61
4	63	GSHTVHYPA	71
5	105	EANAVATLMLHCGVAS	120
6	134	YMTDCAAGLRT	144
7	174	KGHPLIYGGVSP	185
8	192	AGHAFVID	199
9	203	KAGLVSV	209
10	217	VDGYYKIDL	225
11	240	MVRGVYG	246

Contact Pedro Reche

Last Update: 19 August 2022

Figure S1: Antigenic Peptides determinants.

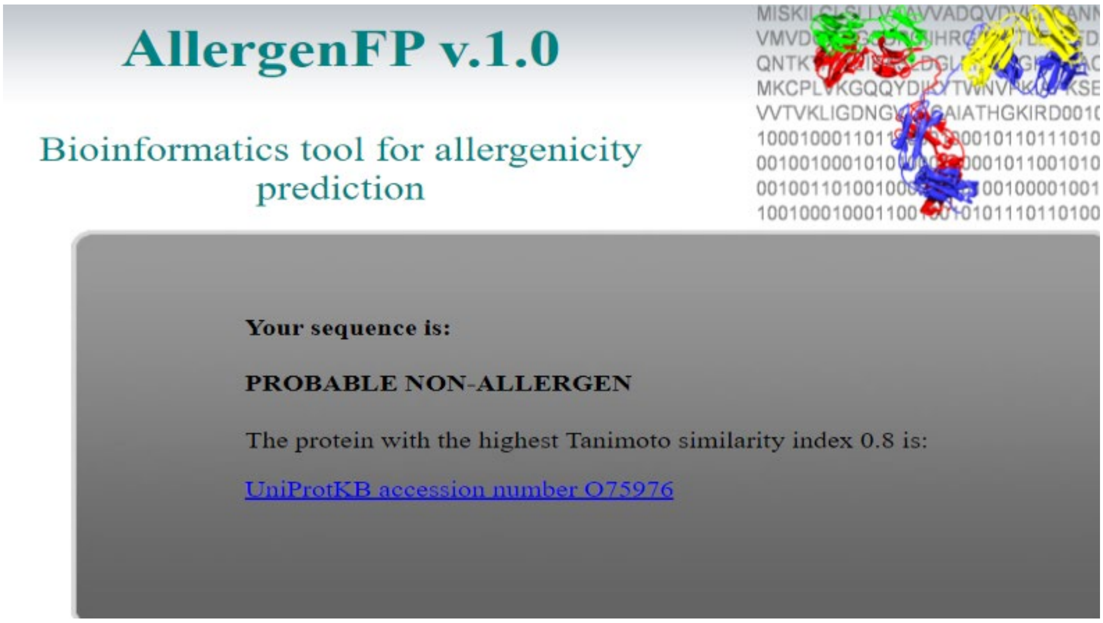

Figure S2. Prediction of Allergenicity.

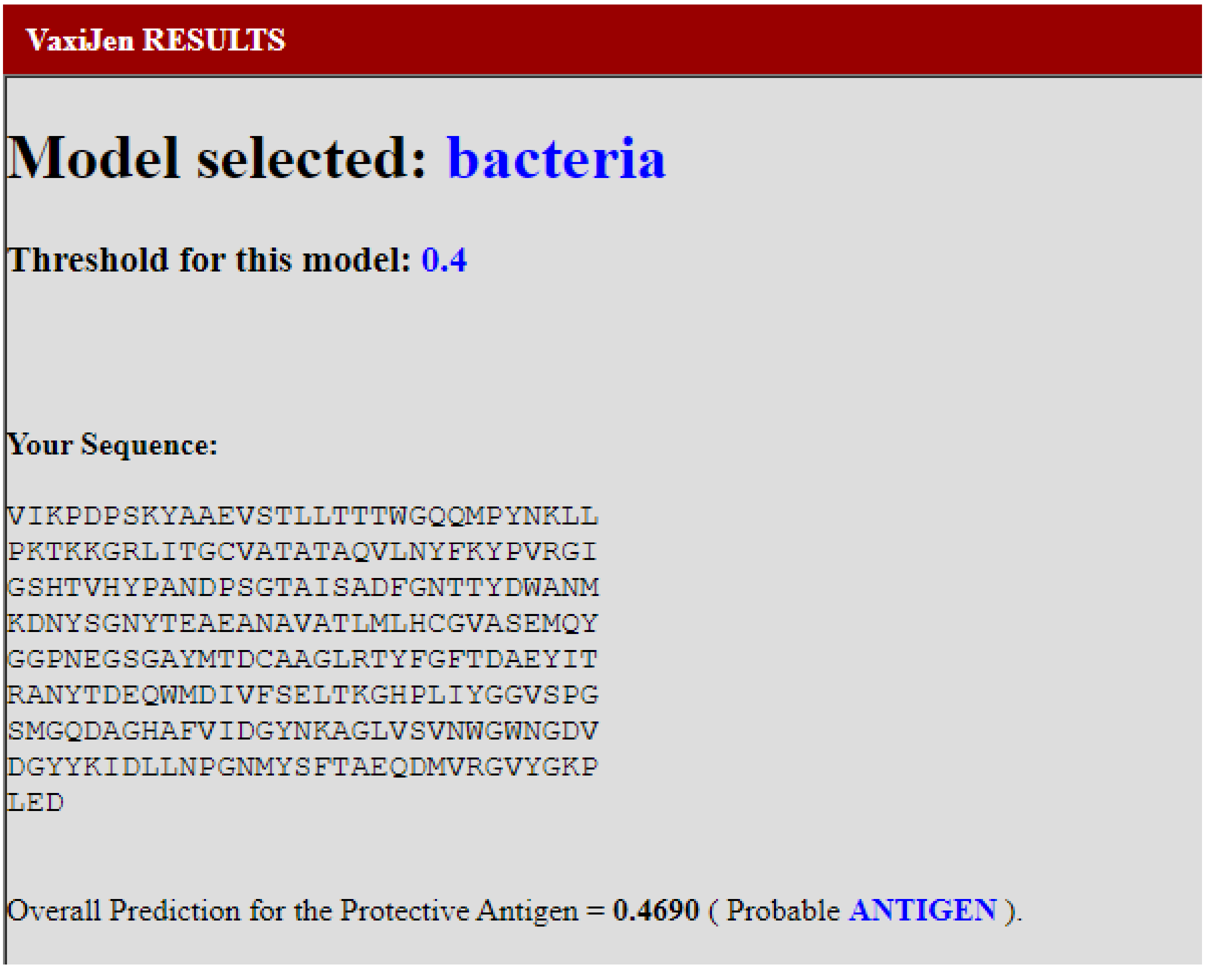

Figure S3. Antigenicity Prediction.

Cluster	Members	Representative	Weighted Score
0	99	Center	-596.9
		Lowest Energy	-661.2
1	74	Center	-566.8
		Lowest Energy	-645.4
2	57	Center	-580.6
		Lowest Energy	-617.8
3	56	Center	-623.1
		Lowest Energy	-651.1
4	52	Center	-542.0
		Lowest Energy	-639.3
5	46	Center	-632.9
		Lowest Energy	-632.9
6	46	Center	-592.0
		Lowest Energy	-611.0
7	35	Center	-612.6
		Lowest Energy	-612.6
8	32	Center	-542.5
		Lowest Energy	-634.2
9	31	Center	-622.1
		Lowest Energy	-622.1
10	31	Center	-594.8
		Lowest Energy	-594.8
[illegible]			

Figure S4: Binding energy of the Predicted vaccine structure with C3.

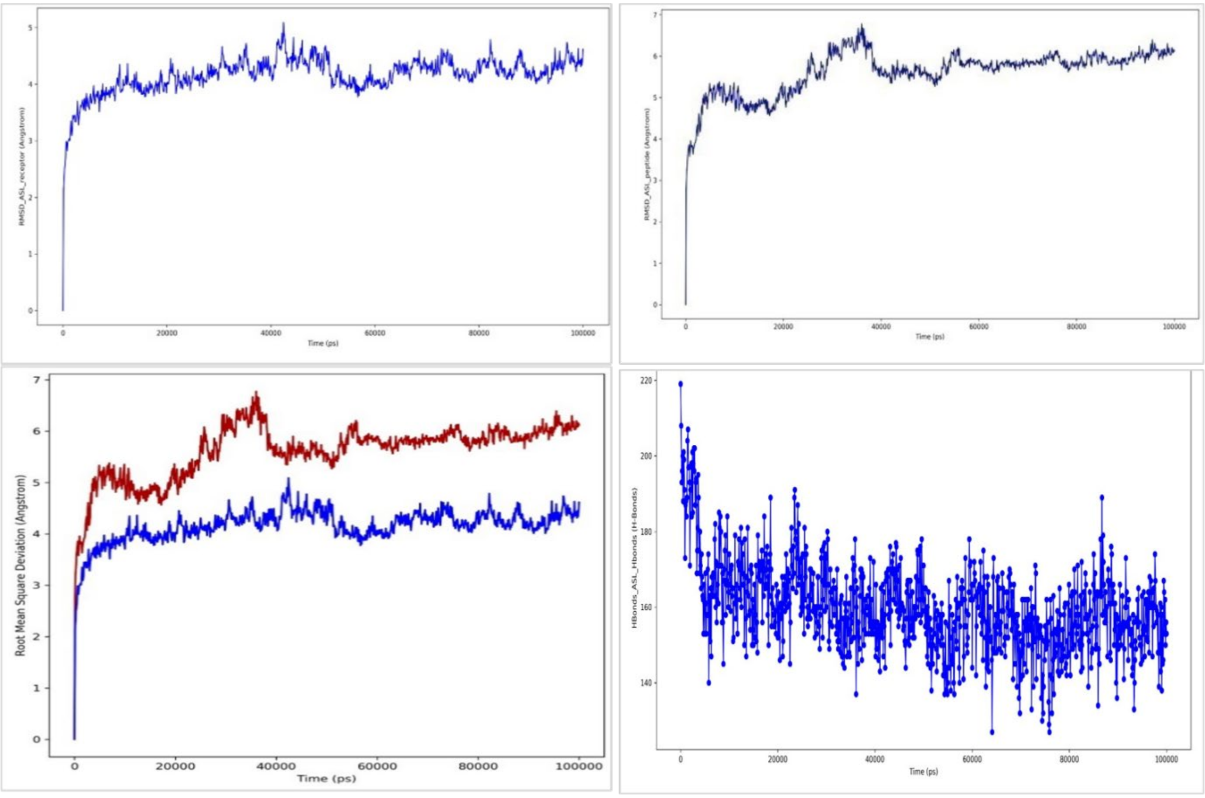

Figure S5: Molecular dynamic simulation analysis.

Table S1: CTL epitopes for N protein (NetCTL1.2).

S. No	Peptide
1.	YAAEVSTLL
2.	TTWGQQMPY
3.	TATAQVLNY
4.	ATAQVLNYF
5.	GIGSHTVHY
6.	SADFGNTTY
7.	MKDNYSGNY
8.	YTEAEANAV
9.	MTDCAAGLR
10.	FTDAEYITR
11.	YTDEQWMDI
12.	WMDIVFSEL
13.	LTKGHPLIY
14.	WNGDVDGYY
15.	EQDMVRGVY
